# Photovoice and Instagram as Strategies for Youth Engagement in Disaster Risk Reduction

**DOI:** 10.1177/10497323221116462

**Published:** 2022-08-06

**Authors:** Christina J. Pickering, Zobaida Al-Baldawi, Raissa A. Amany, Lauren McVean, Munira Adan, Lucy Baker, Zaynab Al-Baldawi, Tracey O’Sullivan

**Affiliations:** 1 EnRiCH Youth Research Team, EnRiCH Research Lab, 12365 University of Ottawa, Ottawa, ON, Canada; 2 Interdisciplinary School of Health Sciences, 12365 University of Ottawa, Ottawa, ON, Canada; 3 LIFE Research Institute, 12365 University of Ottawa, Ottawa, ON, Canada; 4 School of Epidemiology and Public Health, 12365 University of Ottawa, Ottawa, ON, Canada; 5 School of Community Services, 7964 Seneca College, Toronto, ON, Canada; 6 Department of Psychology, 98607 Concordia University, Montreal, QC, Canada

**Keywords:** photovoice, community-based participatory action research, qualitative research methods, youth engagement, youth participation, youth as co-researchers, knowledge mobilization, empowerment

## Abstract

Community involvement is essential for an all-of-society approach to disaster risk reduction. This requires innovative consultation methods, particularly with youth and during pandemic restrictions. This article outlines methods used for a Photovoice project where we brought together student co-researchers from multiple levels (high school, undergraduate, and graduate health sciences) to explore the topic of youth engagement in disaster risk reduction. Over a two-year period, our team used Photovoice as an arts-based participatory method to collaborate with members of our EnRiCH Youth Research Team. We adapted the protocol to continue our project during the COVID-19 pandemic and presented our work in a Photovoice exhibition using Instagram. This article was written from the perspectives of high school and university students on the project. Our hybrid Photovoice protocol facilitated participation through the pandemic, including a virtual presentation at an international conference and online consultation with the Canadian Red Cross.

## Background

The Sendai Framework for Disaster Risk Reduction 2015–2030 was adopted by United Nations (UN) member states and endorsed by the UN General Assembly in 2015, with the aim to significantly reduce disaster risks and losses in all areas of life (UNDRR, 2015). In 2021, the United Nations Office for Disaster Risk Reduction (UNDRR) published the UNDRR Strategic Framework to delineate focused areas to accelerate the implementation of the Sendai Framework for the years 2022–2025 (UNDRR, 2021). One of the priority areas for the next four years includes action in disaster risk reduction (DRR) through partnerships and engagement with stakeholders (UNDRR, 2021). The Sendai Framework stipulates the need for an “all-of-society” approach to engagement and partnership to reduce disaster risks, support community resilience, and leverage power and reach of diverse stakeholders (UNDRR, 2021), including youth groups (UNDRR, 2015).

Youth are one of the populations at highest risk in disasters (Bartlett, 2008; Peek, 2008; UNDRR, 2015). However, youth have limited agency and decision-making power in DRR, and both are needed to move local, national, regional, and global DRR agendas forward (Cox et al., 2019). As such, youth participation in DRR is a relatively new strategy to improve community resilience in disaster management (Amri et al., 2018; Muzenda-Mudavanhu, 2016). As Cox et al. (2019) stipulate, one challenge to youth engagement is ensuring their participation is “more than a checkbox” (p.2), and as such is meaningful and youth-centric. This view aligns with the need to change current dominant top-down models in DRR, in which youth are viewed as passive victims in disasters, rather than active agents of change (T. Mitchell et al., 2008; Muzenda-Mudavanhu, 2016).

Photovoice is a community-based participatory research (CBPR) method in which people take pictures to capture their lived experiences and aspects of their environment, to share with others (Wang & Burris, 1994). As a collaborative research method, Photovoice (Wang & Burris, 1994) is a solution to support bottom-up, meaningful, youth-led collaboration to improve community resilience and adaptive capacity. Photovoice is a method for researchers to use, to support efforts to build community capacity and social capital, thus removing tokenistic youth participation in DRR (Manyena et al., 2008). Capacity building and social capital are key to creating meaningful social change and sustainable community resilience (Poortinga, 2012). Social capital is the impact of social relationships on individual health and wellbeing, public health, and economic development (Szreter & Woolcock, 2004). Photovoice builds social capital by engaging participants as co-researchers and creating opportunities for dialogue with decision-makers.

Collaboration with youth through Photovoice can be time consuming, take greater coordination, and be more difficult than working with adults (Drew et al., 2010; Kramer et al., 2013; Strack et al., 2004). Other critiques regarding Photovoice methods include concern over co-researcher engagement and empowerment (Carlson et al., 2006), power imbalances (Castleden et al., 2008), and meaningful dissemination of results to inform social change (Latz, 2017; C. Mitchell, 2017). We took these important considerations into account during our study by dismantling power differentials, fostering engagement, and ensuring meaningful dissemination of results through youth engagement.

Our study provided a meaningful and youth-centric opportunity for youth to participate in DRR research and contribute to resilience in their community by understanding their perspectives on youth engagement in DRR. The purpose of this article is to provide a detailed account of our methods for a hybrid model of Photovoice (in-person and virtual), including a virtual conference presentation and online exhibition using social media. We also discuss the lessons we learned in building a meaningful opportunity for community collaboration. The findings are available in two separate articles or can be viewed on our Photovoice Instagram exhibition page @yrtphotovoiceproject.

## Methods

### Participants as Co-researchers

Photovoice is a community-based participatory action research (PAR) method (Liebenberg, 2018). In Photovoice studies, participants are viewed as equal collaborators throughout the research process and are referred to as “co-researchers” along with academic researchers (Latz, 2017; Wang & Burris, 1994). In this article, at times when it is important to distinguish between co-researchers, we refer to youth participants as high school students and research assistants/principal investigator as university students/lab director.

### Study Design

Photovoice, as a research method, was developed by Wang and Burris (1994) to enable people to communicate their experiences, community strengths and weaknesses, support critical dialogue about individual and community problems through focus group discussions of visual media, and promote dialogue with policymakers (Wang, 1999). As such, Photovoice combines community-based participatory research (CBPR)/participatory action research (PAR), visual research methods, and qualitative narrative data. Community-based participatory research (CBPR) and participatory action research (PAR) are forms of research in which researchers and communities collaborate and share decision-making with the goal of creating meaningful social change (Israel, 2013; Pickering et al., 2021). Furthermore, visual research methods use visual media (photographs, diagrams, film, etc.) to answer research questions (Rose, 2012), while qualitative narrative data are descriptive oral or written explanations of participants’ perceptions and lived experiences (Latz, 2017). To promote collaboration and social change, and collect photographic and narrative data, Photovoice studies use focus groups as a method of data collection (Latz, 2017; Wang, 1999; Wang & Burris, 1994).

Our Photovoice protocol modified research methods from Wang (1999), and integrated modern community engagement strategies using Instagram, as inspired by Yi-Frazier et al. (2015). Below, in Table 1, we provide a stepwise description of our research protocol.

**Table 1.** Our Photovoice research protocol, modified from Wang (1999).

Steps	Actions
1) Identify objectives and intended outcomes	Discuss study objectives / intended outcomes with youth team members who initiated the project.
2) Submit application for ethics approval	Submit ethics application.
3) Recruitment and information session	Invite youth team members to participate; host parent/participant information session, to introduce Photovoice, outline expectations, and obtain written informed consent.
4) Focus group #1: Facilitated discussion and explanation of first assignment	Orientation for the group; discuss initial photo assignment; review rules on data sources and use of Instagram as a research tool.
5) Take pictures between focus group sessions	Take pictures for photo assignment (high school students).
6) Transcribe audio recordings and create initial codes between focus group meetings	Transcribe focus group recordings and create codes (university students).
7) Focus groups #2 – 9: Member check codes from previous session, present pictures, group discussions, decide next assignment	Review codes from previous session with the team; presentation of pictures by the high school students; group discussion about the pictures and designated topic for the session; reach group consensus on the next photo assignment; plan for exhibition in the final focus group session.
8) Identify themes	Code and identify themes (university students)**.**
9) Refine themes through member checking	Refine themes through member checking with high school students.
10) Plan Photovoice exhibitions with community partners	Distribute invitations, prepare display, host exhibitions.
11) Host Photovoice exhibitions	Present pictures and themes; facilitate discussion with policymakers and community leaders.

### Participant Recruitment

Our study, which spanned 2019 to 2021, was based in Ottawa, Ontario, Canada, and is an initiative of the EnRiCH Youth Research Team (YRT) —a youth-led, grassroots, community-based program offered through the EnRiCH Research Lab at the University of Ottawa (Pickering et al., 2021). The YRT is composed of youth aged 13 and over who participate in research and knowledge mobilization activities in DRR; we use a youth development and mentorship model in which the post-secondary students mentor the high school students on the team. For the Photovoice initiative, four high school students between the ages of 14 and 16 years of age participated throughout the duration of the study. All team members, including the students and lab director, identify as female.

### Identify Objectives, Intended Outcomes, and Ethics

The high school students on our youth research team initiated the conversation about doing a Photovoice project; as such, we had interested participants before establishing our research objectives. Together we discussed study objectives, possible outcomes, and design with the co-researchers prior to ethics submission. We received ethics approval through the University of Ottawa Office of Research Ethics and Integrity.

### Focus Groups and Picture Taking

We held nine youth-led focus group sessions, each two hours in duration, over a period of two years (2019–2020). The format for our focus groups changed between 2019 and 2020 due to the global COVID-19 pandemic. We hosted six focus groups in-person in 2019 and three focus groups virtually using Zoom, in 2020, following a similar format to Wang and Burris (1994):

1) Summarize key themes from the previous focus group (member checking and stimulated recall);
2) Introduce the research question/topic of interest for the current focus group (which was preselected at the end of the previous session);
3) Roundtable presentation and discussion of new pictures taken by co-researchers;
4) Reach team consensus on the photo assignment for the next focus group session.

Prior to our first session, we hosted an information meeting to present the project, the proposed research process, and review the consent/assent forms with parents/parental guardians and high school students. In this meeting, everyone was given the opportunity to ask questions and express their preferences for scheduling the focus groups.

During our first focus group, we reviewed rules around data sources and tools, and came to a consensus on the first photo assignment. Between focus groups, the high school students took 4-5 pictures representing their thoughts, feelings or experiences on the photo assignment. During the focus groups, co-researchers did “show and tell,” presenting their photographs and how they related to the photo assignment (López et al., 2013). In Photovoice, pictures are used to enable participants to explore their worldviews, communicate their ideas to others, and stimulate critical dialogue about issues (Wang et al., 1996). At each session, the high school students presented their pictures and their meanings, followed by an open dialogue about the pictures and photo assignment. At the end of each session, we came to consensus about the topic for the next photo assignment, based on what the high school students wanted to discuss. There were eight people present for most focus group sessions: four high school students who were generating the photos, and three university students and the lab director who participated and helped guide the discussion.

#### Data Sources

Pictures can be used in several ways: co-researchers can take their own pictures, use pictures preselected by researchers, or use pictures, memes, and GIFs from the internet (Latz, 2017). For this study, we limited data collection to visual images taken by co-researchers either on their personal smartphones or digital cameras. One co-researcher decided to use a digital camera, while the other three used their smartphones. As a team, and in consultation with the University of Ottawa’s Office of Research Ethics and Integrity, we decided on the following safety restrictions regarding picture taking: No pictures of other people where persons are readily identifiable; and no pictures of themselves where they are identifiable. In addition to visual images as data, we audio-recorded all in-person and virtual Photovoice sessions. During the sessions, we displayed the images on a laptop for the whole group to see the image being discussed.

#### Instagram as a Tool

In our initial discussions with the YRT about doing a Photovoice project, the high school students asked if it was possible to incorporate Instagram. During the data collection phase, we created a private Instagram account accessible to all team members to facilitate picture sharing and focus group discussions. In contrast to Yi-Frazier et al. (2015), who used Instagram to replace in-person focus groups, our study used Instagram to complement the focus groups during data collection—as well as a venue for our Photovoice exhibition. Before each focus group session, the high school students had the option of uploading their photos to either the private Instagram account or a private Google Share Folder.

#### Strategies to Maintain Focus During Focus Group Sessions with Youth

Studies have found that working with youth on Photovoice projects can be difficult, for example, youth struggle to know what to photograph (Drew et al., 2010; Kramer et al., 2013; Strack et al., 2004). We experienced some difficulty maintaining focus during some discussions. To address this, we provided snacks, pens/paper for drawing, we alternated turns for presenting pictures, and we included time to socialize informally before and after the sessions.

#### The SHOWeD Method

Wang (1999) outlined the “SHOWeD” method for structuring questions—to stimulate conversation during Photovoice sessions. The acronym stands for the following questions:

1. “What is Shown here?”
2. “What is really Happening here?
3. “How does this relate to Our lives?”
4. “Why does this concern, situation, or strength exist?”
5. “How can we become Empowered through our new understanding?” and
6. “What can we Do?” (Wang, 1999, p. 188)

Although the SHOWeD strategy has shown value in Photovoice research to help groups move from superficial discussions to situating images within larger issues (Liebenberg, 2018), it did not facilitate in-depth discussions for our team. Our experience is not unique, with other authors describing the need for different strategies to facilitate Photovoice discussions (Hergenrather et al., 2009; McIntyre, 2003). We found our team worked best in an informal discussion setting, using more natural probes such as: “That is a really unique image - what else can you tell me about it?” or “Thank you for sharing. Before we move to the next picture, does anyone else want to comment?” or “What does this picture mean to everyone else?” or “How would you change ‘x’ issue?”

#### Focus Groups During a Pandemic

We originally planned to host six focus group sessions in 2019, and finish data analysis in 2020. However, during one of our virtual data analysis meetings during the pandemic, the high school students expressed interest in adding more sessions. In 2020, during the first wave of the pandemic in Canada, we received ethics approval to add three more focus groups and modify our protocol to host the sessions virtually using Zoom. We began every meeting with computer cameras on for a quick roundtable, then turned videos off to use the Zoom recording function. The virtual focus groups provided a safe, non-judgmental, space where high school students were able to connect with peers over their fears and frustrations of the global COVID-19 pandemic—and connect at a time when video conferencing was one of the only options to socialize safely.

### Data Management and Analysis

In total, the high school students took over 40 pictures and provided 18 hours of audio recordings from focus group discussions. Analysis was done concurrently with data collection and occurred in four phases: (1) initial coding, (2) member checking for the codes, (3) grouping codes into themes, and (4) member checking for the themes. Initial coding involved a mix of in vivo, inductive, and deductive coding. Wang and colleagues recommend co-researcher engagement throughout analysis, facilitated by summarizing themes from the previous session at the start of each session (Wang, 1999; Wang & Burris, 1994). In our project, after reading each theme aloud, the university students asked if their interpretation accurately reflected the high school students’ discussion and whether there was anything missing. This step provided an opportunity for the university students, who identify as older youth/young adults, to check their positionality through reflexive practice (Braun & Clarke, 2019). After revision of the themes, we finished analysis with 15 umbrella themes and 70 subthemes; these results will be published in two separate articles and can be viewed in full at our Instagram exhibition page @yrtphotovoiceproject.

### Knowledge Mobilization

Photovoice exhibitions are an important output to enable stakeholder engagement with co-researchers, meaningful dissemination of findings, and social change (Wang, 1999; Wang & Burris, 1994, 1997). Our knowledge mobilization activities included an online Instagram exhibition and a youth-led conference presentation at two international conferences (one in 2021 and another in 2022). In this section, we explain how we planned our original exhibition in 2019, and how we adapted exhibitions to an online format. We end this section with a detailed outline of our process creating the youth-led conference workshop and Instagram exhibition, finishing with reflections on direct actions and new opportunities that stemmed from our exhibitions.

### Our Knowledge Mobilization Planning and Implementation

A point of concern in Photovoice research is the meaningful dissemination of findings and how study results are used to inform change (Latz, 2017; C. Mitchell, 2018). Knowledge mobilization is an important final step in the research process—one sometimes missed or rushed. In Photovoice, knowledge mobilization is typically built into the action-based research process in the form of exhibitions and, in our experience, guest lectures or presentations following the project.

### The Evolution of our Hybrid Model Photovoice Exhibitions

In 2019, we used the second half of our final focus group session to plan an in-person art gallery style exhibition in anticipation of using a rented space in downtown Ottawa. We planned to invite influential stakeholders and incorporate interactive booths and discussion tables to enable direct conversation between high school students and stakeholders with the power to create change.

In 2020, when Canada went into COVID-19 lockdowns, we decided on two online exhibitions as our knowledge mobilization strategy; one using Instagram, and another in the form of a workshop. For both events, we collaborated with stakeholders from the Canadian Red Cross, which is an important national disaster response organization—and as such an important stakeholder and knowledge user for our study on youth participation in DRR. We had strong engagement from the Canadian Red Cross throughout the exhibition planning process.

### The International Disaster and Resilience Summit 2021 Workshop

The main purpose of our conference exhibition was to create an opportunity for stakeholders and high school students to talk about action strategies to support youth participation in DRR and public health sectors. We selected a small sample of 10 themes from our Photovoice data to present at the conference. Our interactive youth-led workshop was hosted in collaboration with representatives from the Canadian Red Cross. We designed our 1.5-hour virtual Zoom conference workshop to take the following format:

1. Brief overview of youth engagement in DRR and the EnRiCH Youth Research Team Photovoice Project
2. High school students present a small selection of the Photovoice themes
3. Breakout rooms #1 for direct discussion between students and workshop attendees
4. Full group discussion—debrief on breakout room discussions
5. Brief presentation by the Canadian Red Cross about the importance of stakeholder action on the results of our Photovoice exhibition
6. Breakout rooms #2 with workshop attendees to brainstorm action strategies for youth engagement in DRR in their respective organizations
7. Full group discussion—debrief on breakout room discussions
8. Concluding remarks

For the high school students, it was their first time presenting at an academic conference. To help create a safe space and support capacity building, we scheduled several team meetings leading up to the conference to provide opportunities for the team to ask questions and practice the presentation. We also hosted a practice workshop session with community stakeholders, two days before the actual conference presentation; attendees included researchers and clinicians from Public Health Ontario, PhD students, professors, and administrators—all relevant knowledge users to our study. We received positive feedback from stakeholders during this practice session and experienced productive discussions between stakeholders and youth. As a result, going into the conference, our team felt confident presenting and using technology—and had confirmation that youth voices are valued. During the conference workshop presentation, we engaged with knowledge users from Ministère de la Sécurité publique, Public Safety Canada, and the Canadian Red Cross. Both the practice and actual workshop presentations resulted in important discussions on how to harness the results of our study into action, through policy, practice—and solid commitments and opportunities going forward. For instance, members of the Canadian Red Cross committed to supporting our youth team by peer reviewing our community resource projects (i.e., disaster education modules and children’s book on earthquake preparedness). We also had opportunities to further present our work since the workshops, such as presentations on youth engagement in DRR to new members of the Canadian Red Cross; graduate students in a research methods course; and an international conference in 2022, in which two high school student co-researchers presented by themselves on behalf of the team to an international audience of health promotion stakeholders.

#### Maximizing Participation Through Support and Mentorship

For the virtual conference workshop, the breakout room discussions were hosted by three members of our team (a high school student, a university team member, and a member of the Canadian Red Cross). The high school students facilitated the discussion with workshop attendees—with the understanding that the university team member and Canadian Red Cross representative were there to support them. The members of the Canadian Red Cross participated in the workshops in dual roles, as both co-facilitators and important knowledge users/stakeholders in the field; they participated actively in all discussions, bringing in examples from their own organization and potential action strategies.

### The Instagram Photovoice Exhibition

The social media expertise and experiences of the high school students were integral in our decision to use Instagram as a platform for our virtual exhibition. Their expertise were essential in the design of the account and Instagram posts. Our Instagram post development process consisted of five steps: 1) Designing the Instagram posts in Canva, 2) creating the Instagram account, 3) scheduling posts, 4) editing the posts and captions, and 5) posting the prepared content/pre-determined hashtags and tagging accounts.

To create the Instagram posts, we used a free graphic design platform called Canva to help convert the thematic results for an Instagram audience. We created a group account to centralize our posts. For project feasibility, we designed a template to standardize the aesthetic of the account posts. Every team member selected one to four umbrella themes to translate into conversational style Instagram posts using the Canva template. The template had the following standardized design elements to ensure clarity, ease of reading, and to capture the audience’s attention:

- Simple coloured outer border with a thinner inner border.
- Every first slide consisted of a theme title, a subtheme title, and the associated photo.
- Standardized and readable font and font size.
- Themes explained within the posts, as opposed to the captions.
- Use of white space, short explanations, and conversational language.
- Quotations displayed in italics.
- Bold font and underlining to emphasize key words.
- Free graphics (e.g., a graphic of a mask, or youth celebrating) to provide visual aid.
- Every post concluded with a final slide that read “Join the Discussion” to encourage followers to reflect on the information presented.
- The Photovoice team logo on every slide.
- Preselected colour scheme to ensure each post was aesthetically complementary and professional.

**Figure 1.**
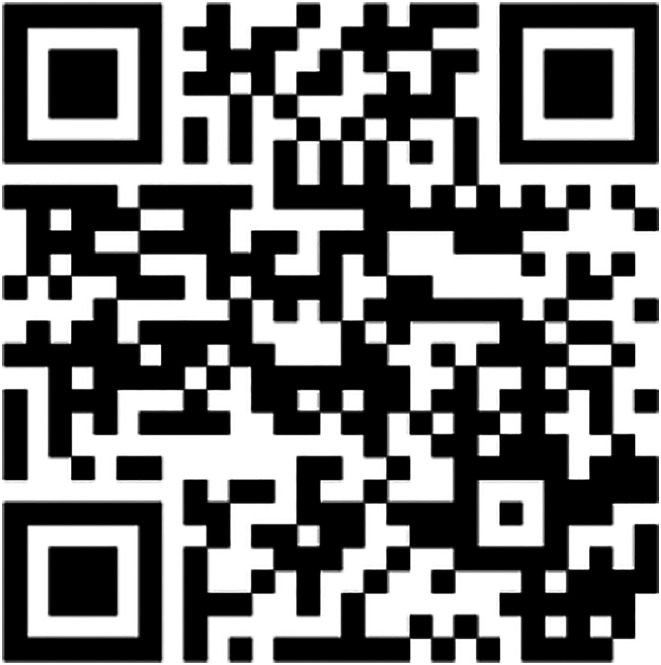
QR code for the EnRiCH Youth Research Team's Photovoice Instagram Exhibition.

Designing the Instagram account itself was another important step in creating our social media exhibition; the business account features enabled us to see the metrics of our audience (gender, location, age group, etc.). We chose the username @yrtphotovoiceproject because it is memorable and can be associated quickly with our project. During the exhibition, our team followed a schedule that consisted of posting once daily. During the week of the International Disaster and Resilience Summit, we increased our posting to twice a day to build on increased viewership resulting from the conference workshop. Tagging other accounts was essential to ensure relevant organizations and stakeholders would receive notifications about our post. Hashtags also helped our posts appear in searches on Instagram, thereby enlarging our reach and visibility online. Finally, we also developed a stakeholder engagement strategy for interacting with other Instagram accounts throughout the exhibition. Figure 1 is a QR code, which upon scanning with a cellphone camera, will take readers directly to our Photovoice Instagram Exhibition.

It is important to note that virtual exhibitions, while extending reach, are limited by the current state of technical advances, technical problems, and a lack of control over who attends and interacts with the posts. For the conference, we were unsure who would attend and the number of participants to expect. We had a plan to adjust our conference format for larger or smaller numbers. For the Instagram exhibition, while we could see how many people were interacting with our page, there were limited interactions on each individual post. However, the platform provides an accessible location for people to view the digital exhibition, which gives it exposure over a longer time period (e.g., when we refer to it in our presentations).

## Reflections and Lessons Learned

In this section, we reflect on important elements of our Photovoice protocol, such as emphasizing community voices, building capacity, and social capital. We also reflect on lessons learned, such as creating a safe space, and using Instagram as a tool.

### Emphasizing Community Voices

Throughout our project, we emphasized and maintained a co-development and partnership process (Knowledge Institute for Child and Youth Mental Health Addictions, 2021). The high school students noted it was empowering to feel engaged as co-researchers. They described their previous experiences with other organizations engaging youth, where it felt consultative—or tokenistic—and they did not enjoy this type of collaboration. Shared power in decision-making for this Photovoice project was one of the most important and valued aspects highlighted by high school students; it was integral for their motivation to participate, sustained enjoyment of the collaboration, and feeling fulfilled, empowered, and meaningfully engaged.

### Building Capacity and Social Capital

To support empowerment and engagement in Photovoice research (Carlson et al., 2006) and protect against power imbalance (Castleden et al., 2008), we emphasized equitable reciprocity through youth development and capacity building. The high school students benefitted from 1) equal opportunities for decision-making and control as co-researchers, 2) learning qualitative research skills, and 3) opportunities to build their resumes/cv’s (i.e., co-researcher on a Photovoice study, co-creators of an Instagram Photovoice Exhibition, co-authors on three peer-reviewed manuscripts, presenters at a conference, and guest lecture panelists). Much of the strength behind our Photovoice project stemmed from strong bonding social capital amongst our team, supporting bridging social capital with other youth-led community organizations on Instagram, and building linking social capital through sustained collaboration with the Canadian Red Cross.

### Creating a Safe Space

Photovoice has the potential for power imbalances in the research context (Castleden et al., 2008). Knowing this, our priority was to create a safe environment to support co-researchers working together effectively (Wang & Burris, 1997). We were careful to avoid the classic interviewer/interviewee power dichotomy. Our strategies to create a safe space included: (1) setting clear expectations and boundaries regarding equal collaboration, (2) eliminating structural barriers to participation (e.g., geographical, monetary, and time barriers), (3) transparency about informed consent, (4) selecting a familiar meeting space (i.e., our research lab in downtown Ottawa), and (5) providing time to socialize informally before and after meetings.

### Instagram as a Tool

Although Instagram worked well as a virtual venue for our Photovoice exhibition, it did not work well as a data collection tool. We offered the option of posting on our private account between sessions, to share photos and comments, but there was little co-researcher engagement on the account and they explained it was cumbersome to switch between their personal accounts and the group account. In a future project, we would use Google Shared Folders as our data collection tool and use Instagram solely for a virtual Photovoice exhibition.

## Conclusion

Photovoice is an engaging qualitative research method to include community members historically excluded from DRR (Wang & Burris, 1994). Important considerations for using this method for youth engagement includes dismantling power differentials, fostering engagement, and ensuring there is meaningful dissemination of results. The hybrid Photovoice protocol we described here was useful for addressing some barriers to traditional in-person implementation and maintaining momentum during the context of pandemic lockdown. For instance, the accessibility offered through a hybrid Photovoice protocol can be leveraged to support inclusion in DRR initiatives (e.g., for persons with disabilities or language barriers, neurodivergent individuals, geographic limitations, and caregivers).

Instagram provides a suitable exhibition space for knowledge mobilization, with its broad reach—but must be accompanied with engagement strategies to maximize impact. Future research could build on this method by incorporating Instagram and other forms of social media (e.g., Tik Tok) into Photovoice research, or other virtual formats for exhibitions (online virtual art gallery space, YouTube, etc.). Further, using social media helps support a youth-centric approach to participation in DRR and thus enables meaningful engagement.

Our hybrid Photovoice model, with an emphasis on youth development, is a strong method to foster meaningful community engagement and stakeholder partnerships to reduce disaster risk and losses and mobilize the all-of-society approach set out by the Sendai Framework. Further implications for DRR include providing youth, and other groups historically excluded from DRR, with an environment that supports their agency and provides them with decision-making power to move local, national, regional, and global DRR agendas forward in a way that reflects the needs and assets of the community.
